# Address to the Graduating Glass of the Ohio College of Dental Surgery

**Published:** 1917-06

**Authors:** N. S. Hoff


### ADDRESS TO THE GRADUATING CLASS OF THE OHIO COLLEGE OF DENTAL SURGERY

BY N. S. HOFF, D.D.S., JUNE 1, 1917

I shall ask your indulgence while I allude very briefly to some events in the development of the dental profession that may serve as a basis for a few observations which I trust may merit your consideration because of the felicitous circumstance of this occasion. I am going to take a chance on overtaxing your patience by rehearsing some historic events that doubtless are quite familiar to you all.

It should need but slight reflection to convince anyone who has made a study of the origin and progressive development of the art, and science, of dentistry that it is a much simpler task to trace the story of dentistry backward from our own times, than to place one's finger on the exact spot on the map where it first happened. In fact, we have no records that authentically inform us of dentistry as a special practice in the early history of the old civilizations. Hippocrates, the great medical writer of Greece, about 400 B. C., tells us there were doctors in Egypt in his time who specialized in treating the teeth, and his observations are confirmed by the wonderful ''papyrus'' found by Professor Ebers in a very old Egyptian tomb, which it is claimed dates back more than 1500 years B. C. In this old document many dental remedies are recorded but no technical restorations are suggested. From the time of Hippocrates dental art can be traced with considerable definiteness through the middle ages and the period of the Renaissance, up to about 1700 A. D. when we find that it begins an artistic and professional existence in France from whence it crossed the seas to England and the United States, in which latter country it seems to have discovered an environment most favorable to its development.

We shall not weary you with detail of this early experience except to say that fortunately certain men with large mental and moral equipment became identified with the practice of dentistry as a means of livelihood and foresaw a destiny which caused them to devote their lives to its realization. I can not here even enumerate their names. For our purpose I wish only to say that they were not only men with large visions, but they had ideals that were so fine that they threw all their energies into their efforts to realize them. They foresaw that it was necessary to have an educated profession in addition to a class of skilled artisans. Consequently they organized a college for the profession; they also established literary communication among the members of the profession. The Baltimore College of Dental Surgery and The American Journal of Dental Science were established in the eastern section of this country in 1840, and the Ohio College of Dental Surgery and The Dental Register of the west for all the great Mississippi valley. Who can tell what would have been the career of dentistry had not Chapin A. Harris and Horace Hayden become interested in dentistry in Baltimore, and had not James Taylor located in Cincinnati instead of in Vicksburg or Crawfordsville? Possibly we should not be in this place at this time.

Before the colleges and journals came, dentistry was not a profession; but there were fortunately a few men with professional ideals, and those few saved us from the narrow and selfish ideals of the commercial artisan. The college took the apprentice out of the office of the uncultured preceptor and gave definiteness and comprehension to his training, which before had been too frequently chaotic and unskilled.

Our first dental faculties were three-fifths medical science and two-fifths dental art teachers, and a larger proportion of dental teachers in the beginning were also medically educated than is now the case, for the reason that a knowledge of the human body and its diseases has always been thought fundamental to the practice of dentistry as a special branch of the healing art. In the later development of our system of education, while not minimizing the medical science topics in our college curriculum we have been emphasizing what we have termed the technical phases of dental education more especially. In the very recent years there are some indications of a closer study of the medical science subjects; not, however, to the detriment of the technical subjects, but that there may be a more harmonious balancing of the course, with the resultant stimulation of a wider culture and a higher order of skill in the science and art of practice.

If we were to compare the amount of instruction given in our dental colleges a half century ago with the present time we could, perhaps, get a better idea of the advancement we have made, and we should have every reason to expect present day graduates to exert a greater influence than ever before, because of this broader and stronger teaching effort. Then the course was for four months and only two sessions; now it is to be eight or nine months and four sessions. An increase of more than three times as many subjects and more than four times as much time spent on the curriculum.

When Harris asked the medical college in Baltimore to add dentistry to the medical curriculum he was informed that it was not worth while to teach dentistry in a medical college. Now the highest medical authorities in the land are saying that without dental assistance some of the most troublesome systemic diseases can not be cured.

All of which goes to show that dentistry has made substantial advancement and that its founders and builders had the right ideals, and they have materialized into a distinct professional calling that is coming to be recognized as one of the most useful and honorable of the professions.

We might also call attention to the fact that the men entering dental colleges today are required to possess a better preliminary education than ever before; and more teachers than ever before are devoting their entire time to imparting dental instruction, instead of making such teaching merely an incident of their professional careers. The fact that we have better educated students to start with, and faculties of trained specialists as instructors, should produce a better product; and the future of the dental education, it seems, should be more promising in ideal results than at any time in our history. These facts should react on professional advancement in general. It is well known that practically all our college textbooks are now made by college teachers, and the majority of our current literature comes from the same sources. There are, however, evidences of greater interest in general literary work by the general and special practitioners than ever before. May we not anticipate a still greater interest in this direction in the future because of the better educated dentists?

With this historical review so briefly presented, what significance has the past and up to the present development of the profession to do with the new men who today are entering its ranks?

We wish today that, like the runners in the relay race, we could reach out our hand and touch yours with all the power and magnetism of inherited traditions and personal experience and send you off with a momentum that would speed up all your energies to their full capacities, so that when you have finished your lap you shall have a larger view and a much greater achievement to pass on to your successors. The advantage the forerunner in the relay passes to us is not usually appreciated as it should be, we observe with regrets. It is true that some men are satisfied to live on their inheritance and die perfectly contented with just having kept the faith.

I know of no more helpful stimulant to progress than the study of men who have accomplished worthy things, historic, if necessary, but first-hand contact preferably.

One of the most eminent teachers and writers of our time told me he got so interested when a young man reading discussions of dental topics by eminent men as reported in the dental journals, that he left his home town and went to a large city, where some of these men lived, and began a practice, that he might know them personally.

Environment and personal associations undoubtedly do much to keep us lined up with progressive ideals. It may be trite for me to say that a good father and a devoted mother, a true friend, or good books will do much to keep a man in the straight and narrow way, but I wish I could impress you as strongly as I feel, that the true and progressive men in the profession you are entering will be of such incalculable help to you in the development of professional ideals that you *must* know them, and you *must* open your minds and affections to a sympathetic understanding of those things which have made them honored and useful men in the profession.

I do not ask you to become mere hero worshipers, nor to ally yourselves with men who have accidental power or influence; but you can easily pick out of the crowd the real men with high ideals who are worthy of your confidence. As I stand here before you my memory goes back to this day, forty-one years ago, when I received my diploma from this historic institution, and I can recall gratefully the men who gave me my instruction. Most of them have ceased their work here, but so long as memory serves me, I shall look back to them with love and esteem because of their many kind and helpful words of advice and counsel which have guided and helped me to see my duties more clearly before me, and they have continuously urged me on to better service for the cause which has so largely absorbed my life. I wish I had time to tell you about these men, and others that I have since known and learned to honor and love, as I am sure I could give you food for thought that would give you new hopes and courage to throw your lives wholly into the work that is now before you.

It seems to me that we had bigger and more famous men to admire and follow when I was a young dentist; but possibly it is because giants are so common today that they are not so conspicuous. We have the giants of former times in our literature which I most earnestly commend to you, and you will meet up with new ones at every gathering of dentists in conventions and in social gatherings. Get acquainted with these men personally, if possible, but get their viewpoints and study them for yourself. Catch the spirit which animates them as it may be the spark that will ignite you.

We mourn the loss of Miller and Black, and I have heard men say there are no successors in sight for these men. I can't believe it; because they were not unusual as men go. I knew them both rather intimately, and two more simple natured men I never met. Yet they both were great, possibly the greatest scientists our profession has ever produced. They were both filled with the idea that they must do something for suffering humanity, and they spent their lives without reserve or hope of reward to find a practicable cure for dental caries. I should not be surprised if it developed that there was another Miller or a Black in this graduating class. If it should prove so the faculty of this school would be compensated for its labors and sacrifices and the world will be happier and better because you have made good in your calling.

I am making these observations that I may lead up to some thoughts which I think may be more helpful to you in realizing in a measure the great service you are to be called upon to render to mankind.

### OUR CALLING

The dental profession is being called today to a more exalted service than our founders ever dreamed of. In fact, a challenge has been served to us by one of the leading medical authorities, which implies that we must qualify or abandon our assumption of being a beneficent profession, or a branch of the art of healing.

This challenge has been discussed or restated by almost every address given by the presidents of our various dental societies during the past two years, but as yet no one has come forward to defend our cause by clearly outlining the nature of this great step. I do not know what this step may be, and in fact, am not seriously disturbed by the defy, as there are some very important and more practicable affairs that seem to demand our immediate attention. It might be highly desirable for us to make some great discovery that would add materially to our resources for alleviating human suffering and that would justify our sentimental claims to greatness, but in my judgment it is more necessary that we, as a profession, may be imbued with those ideals that have so far made our calling respected and honorable. Our traditions are comparatively so new that they may not possess the same powers to transform our ideals or reform our characters as those of the older professions, and yet it may be an advantage that we are not restricted by traditions which sometimes restrict the clearness of our visions. There are, however, enough precedents to supply motives that should appeal strongly to every young man who enters the dental profession, and give him a determination to do his best to realize all the opportunities for service that may legitimately come to him in the practice of his profession, that he may uphold its dignity and contribute something towards its advancement.

Each of your teachers had doubtless done the best he could to assure you that his particular subject in the curriculum was the most important, else he has failed measurably in his office. You may have been tempted, as many students are, to concentrate your life's work on some special department of the profession, either because the whole seems too large, or you may have become attracted to some particular line of work. This may be because your instructor had a faculty of impressing it upon your mind, or because of his skilful demonstrations or beautiful word pictures. There are substantial arguments for graduates entering upon these special lines at once, but the average man will select his field of work more accurately after some experience with the requirements of general practice on his own responsibility.

The importance of selecting a place of practice that will meet one's ideas of practice is too often lightly considered. It is often the result of accidental circumstances rather than of deliberate consideration. One can do business in a small village, a good sized town, or the largest city of the land, just as he elects; but it is rather important that he consider carefully his own characteristics and attainments that he may not be a misfit, and so lose his chance of developing professionally to the type of professional service that will provide the proper stimulant for his largest development.

Our profession does not offer opportunities for the accumulation of great wealth or for spectacular careers, neither are honors so conspicuous or so easily attained as in some other callings. Few professions, however, offer so great a variety of opportunities for personal service of value, and of vital consequence to humanity. Few offer greater opportunities for special culture in collateral sciences, the arts, or in social relations. If you have tastes for certain things that are obtainable only in definite localities these should be considered first in choosing a location as contentment means much for successful service and personal development.

The field is unlimited and every climate and place is open to you. The need for your service is unlimited and you have only to possess the ability to meet the demands to secure the rewards for merited service. For the most part the pioneering has been done for you, and the more fully you meet the requirements the more certain your success.

It may not mean much to you if I say that eighty per cent. of the people of this country need dental services which they do not get, and the other twenty per cent. are not fully served; *because,* you do not think of your problems in the light of mere statistics. Perhaps it may be more impressive if I tell you that every registered dentist in the United States should take care of the teeth for 2100 people of various ages and social conditions. This means that he will only be able to serve each one of these 2100 people for one hour each year, providing they should all apply. This estimate is based on the assumption that the dentist will be in his office seven hours each day for 300 days each year, which is a fair average of time one should spend in actual practice. This estimate, of course, is impracticable, because 1600 of these patients will never apply for service, but if only twenty per cent., or 400 should apply, one should need to do a whole year of work for each of them in less than a day. It would be almost as much as a dentist could do to keep the teeth of his patients decently free from deposits with no more time at his disposal, to say nothing of the usual repair work needed.

The dentist today who is fully serving 300 patients with modern dentistry has about all he can do to advantage. And according to the figures given above he is doing only one-seventh of his allotment. All of this should show you rather clearly that your field of opportunity is not restricted. As a matter of fact, the people of this country are becoming more intelligent every day, as to the value of their teeth, and are insisting on better professional care than ever before, and the obligation which our profession must meet is to take care of these people on our present basis of practice, or devise some method of curtailing the necessity for so much service of the present technical character.

It will be evident to you that you have made no mistake in choosing a calling where all your energies will be required and in which there is a wide opportunity for personal development and usefulness.

To show you further what there is before you in the way of a career that will tax your physical and mental energies, and if adequately followed will lead to the cultivation of a noble character, I want to add some additional details that may give you a better view of the field of opportunity that indicates a greater career of usefulness than we can at present realize.

For many years our service has largely taken the form of relieving suffering and repairing the losses caused by the increasing prevalence of dental caries, and its consequent more serious oral sequellae.

We have confined our attention closely to the immediate dental environment, and it seems that we may soon reach the acme of perfection in the art of restoring to practically normal efficiency the functions of mastication and speech by the refinement of our technic in operative prosthesis. By the great improvements made in pathology and surgical treatment of dental diseases our care of these cases seems to be under practical control except in a comparatively few cases of deferred treatment which leave no basis of recovery, or where systemic complications preclude adequate local interference. In fact many restorative procedures are now definitely certain of successful treatment which a few years ago were considered hopeless.

By prophylactic procedures we now to a large extent interrupt or at least inhibit progess in the development of dental caries and peridental diseases which formerly were subject to operative treatment or to inevitable loss.

By improved methods and more skilled technical procedures we are now replacing lost dentures with substitutes that give the patients a larger degree of comfort and efficiency than ever before.

By orthodontic procedures we are not only correcting many of the unsightly malformations of the features, but increasing the nutritive functions to the general benefit of the entire system. We have learned that diseases of the oral cavity have a direct as well as a reflex influence on the general systemic functions, and that many physical ailments which physicians have failed to cure respond to local treatment. These diseases and their systemic influences are now being studied clinically and scientifically and it seems probable that we shall have to assume new responsibilities of a more serious character than we have been accustomed to deal with, and this will probably modify to some extent our technical methods. This may mean that the dentist of the future should be a better educated dental physician to cope with his opportunities and his enlarged responsibilities.

Preventive dentistry in the direction of the development of the typically normal, or physically perfect man is perhaps the greatest achievement that may fall largely to the active cooperation of the dentist with the physician. The study of the etiology of disease has led us into the most intricate study of the nature of infection, and the most careful scientific study has so far failed to develop specific treatment for the more serious diseases which afflict the human body. And the belief seems to be swinging back to the old idea of vital resistance as the most important factor in general prophylaxis. If this comes to be generally recognized it seems evident the nutrition of the developing body is the essential and predetermining factor in insuring good health. As nutrition begins with the oral and nasal functions the importance of a correct dentition and a normal respiratory tract is very evident. This involves a more careful dental supervision of the development of the first dentition and the maxillary environment from the earliest practicable period of the child's existence. It will also mean a closer and more accurate study of the physiology of developing organs of the mouth and respiratory tract by the dentist and rhinologist as well as the whole question of nutrition and hygiene. This, you see, opens up a field of observation and service that will greatly increase the demand for dental service, and should, as time goes on, decrease the necessity for so much technical art work; at least for the young, and should defer prosthetic restorations to a much later period of our patients' lives than is now customary.

It may be that this is the next great step in preventive medicine which Dr. Mayo predicts is to be taken by the dental profession. If so, it will require the same concentration of effort that has characterized the profession's development in its technical art, and a more profound study of the sciences involved than we have heretofore given to these departments of our profession.

This is too big an undertaking for any one or a few men to demonstrate, but it is not too much to hope that it may be taken up by the profession in general with such careful study and clinical observation as may be possible, and that practical results will greatly improve oral and facial development, and that malocclusion and caries of children's teeth shall disappear as disease producing factors.

Is there anything you can think of that would bring greater happiness into this world than the development of the physically perfect man, and do you not believe that there is a fair field here for you to enlist your talents and energies for the greatest benefit to humanity possible? I

am sure if our profession shall be permitted to contribute materially to this beneficent objective we shall all be grateful to have had so great a share in the world's regeneration.

Now this may be a larger vision than I am justified in bringing to you at this time, but I am so confident that it will some day be realized that I trust you may be permitted to have a large interest and share in its accomplishment.

In closing, I wish to welcome you to all the history and traditions of a most honorable and worthy profession, developed by noble, self-sacrificing men, who saw their visions and have started us on a career of useful living with a momentum that assures a successful career if we can follow the trail and hold fast the best traditions of a calling which will demand the very best there is in each one of you. That you may qualify in a large measure is our earnest desire.

